# Cognitive Ageing in Africa: Systematic Review and Meta-Analysis of Pre-Dementia Prevalence and Associated Factors

**DOI:** 10.24248/eahrj.v9i2.840

**Published:** 2025-12-24

**Authors:** Samwel Sylvester Msigwa, Jackline Zambi, Maiko Charles Mkwambe, Elizabeth Mareale

**Affiliations:** a Department of Psychiatry and Mental Health, School of Medicine and Dentistry, The University of Dodoma, Kikuyu Ave, Dodoma, Tanzania; b Department of Research and Training, Mirembe National Mental Health Hospital, Hazina, Dodoma, Tanzania; c Kairuki University, Chwaku Street, Dar es Salaam, Tanzania; d Ministry of Health, Government City-Mtumba, Dodoma, Tanzania; e School of Medicine and Dentistry, The University of Dodoma, Kikuyu Ave, Dodoma, Tanzania.

## Abstract

**Background:**

Africa is experiencing rapid population ageing, yet the prevalence and determinants of pre-dementia stages – mild cognitive impairment (MCI) and subjective cognitive decline (SCD) – remain poorly defined. Global reviews include only a few African studies, limiting reliable estimates and obscuring regional risk factors. This study provides the first comprehensive synthesis of pre-dementia prevalence and associated factors across Africa.

**Methods:**

We systematically searched PubMed, Embase, Web of Science, and African-specific databases from inception to March 2025. Studies reporting MCI or SCD prevalence in adults aged ≥50 years were included. Data were pooled using random-effects meta-analysis. Subgroup analyses examined heterogeneity by diagnostic criteria, setting, and study features. Meta-regression assessed links with demographic and cardiometabolic factors. Study quality was evaluated using the Joanna Briggs Institute prevalence checklist.

**Results:**

Twenty-eight studies from nine countries included 26,067 participants and 5,924 cases (SCD: 2,318 participants, 1,162 cases; MCI: 23,749 participants, 4,762 cases). Pooled prevalence was 50% (95% Confidence Interval [CI], 40 to 59%) for SCD and 20% (95% CI, 14 to 28%) for MCI. Heterogeneity for MCI was extreme (I^2^≈99%), mainly due to methodological variation. Studies using neuropsychological cut-offs alone reported nearly threefold higher MCI prevalence than those applying clinical criteria. Meta-regression identified associations between MCI prevalence and hypertension (β=.052; P=.017) and diabetes (β 0.053; *P*=.024).

**Conclusion:**

Pre-dementia stages are common in African older adults. Methodological variation underscores the need for standardized, culturally validated criteria. Cardiometabolic comorbidities are key modifiable risks, supporting integrated interventions for cognitive health in Africa.

## BACKGROUND

Population ageing is accelerating globally, with low- and middle-income countries (LMICs), particularly in Africa, experiencing the most rapid demographic shifts.^1,2^ This demographic transition is accompanied by a rising burden of age-related cognitive disorders, including dementia.^3^ Mild cognitive impairment (MCI) and subjective cognitive decline (SCD) represent critical pre-dementia stages, offering opportunities for early intervention to delay or prevent progression to dementia.^4,5^ The definition of MCI is measurable cognitive deficits that do not substantially impair daily functioning, while SCD reflects self-reported cognitive decline in the absence of objective impairment.^4–6^ Accurately characterising the prevalence and risk factors of these conditions is essential for public health planning and clinical intervention in Africa's ageing populations.^7^

Despite global advances in dementia epidemiology, Africa remains markedly underrepresented in research on cognitive ageing.^8^ Previous systematic reviews and meta-analyses, including Bai et al,^9^ with four African studies, McGrattan et al,^10^ with eight, and Salari et al,^11^ with seven, incorporated very limited African data. This underrepresentation partly reflects the fact that African research is less likely to be indexed in major international databases compared to African-specific repositories, making it harder to capture in global evidence syntheses.^12,13^ As a result, regional estimates are unreliable, and subgroup analyses are underpowered, failing to reflect the continent's demographic, cultural, and methodological diversity.^9^ Notably, no prior review has comprehensively synthesized evidence on SCD in Africa, leaving a significant knowledge gap regarding this prevalent early stage of cognitive decline.

Africa's context, including high burdens of infectious and cardiometabolic diseases, lower educational attainment, and limited healthcare infrastructure, likely shapes pre-dementia risk and presentation of pre-dementia conditions.^8,14^ Methodological heterogeneity across studies, such as inconsistent diagnostic criteria and non-standardized neuropsychological assessments lacking cultural or educational adaptation, further obscures the true burden of MCI and SCD.^15,16^ These challenges impede reliable, comparable prevalence estimates and limit the evidence base needed to inform policy and interventions.

To address these gaps, we conducted a comprehensive, Africa-specific systematic review and meta-analysis of MCI and SCD. Our objectives were to: (1) generate robust pooled prevalence estimates for MCI and SCD across African populations; (2) explore sources of heterogeneity, particularly diagnostic criteria, through detailed subgroup analyses; and (3) identify demographic and cardiometabolic risk factors using meta-regression. By providing a continent-wide synthesis, this study establishes a definitive evidence base to guide standardized research, inform clinical practice, and shape targeted public health strategies for cognitive health in Africa.

## METHODS

### Search Strategy

In this systematic review and meta-analysis, we searched PubMed, Embase, and Web of Science from January 1966 to March 2025, corresponding to the earliest inception date among the databases searched. To enhance African specificity, we supplemented these searches with African Journals Online (AJOL). Additionally, forward citation tracking and backward reference searches were conducted iteratively until no additional studies were identified. Search terms included keywords and Medical Subject Headings (MeSH) for “mild cognitive impairment,” “mild neurocognitive disorder,” “cognitive dysfunction,” “subjective cognitive decline,” “older adults,” and geographic identifiers such as “Africa,” “Sub-Saharan Africa,” and individual country names. Forward citation tracking and backward reference searches were conducted iteratively until no additional studies were identified. The full search strategy is detailed in Tables S1–S3.

The study protocol was prospectively registered on PROSPERO (CRD42025646699) and is reported in accordance with PRISMA guidelines.^17^ Methodological filters were applied to identify observational studies – cross-sectional, cohort, or baseline data from longitudinal studies – reporting prevalence estimates of MCI, cognitive impairment no dementia, or SCD in community-dwelling or primary care populations. No language restrictions were applied during the search, however, only Englishlanguage studies were included due to translation resource limitations. References were exported to Covidence (Veritas Health Innovation, Melbourne, VIC, Australia) for deduplication and screening, with additional duplicate removal using EndNote version X7 (Clarivate Analytics, Philadelphia, USA). Two reviewers independently screened titles, abstracts, and full texts, with disagreements resolved by discussion or consultation with a third reviewer. For studies reporting overlapping populations, the publication with the largest sample size or most comprehensive diagnostic data was retained.

### Eligibility Criteria

Eligible studies included adults aged ≥50 years and reported prevalence of MCI or SCD based on validated cognitive assessments or self-/informant-reported memory complaints, with sample size, age, and sex distribution reported. Studies were conducted in community-based or primary care settings in any African country. Inclusion required original prevalence data using subjective cognitive complaints combined with objective cognitive testing or standardized study-specific criteria. Subjective memory complaints were reported by 46% of participants, while 16.9% demonstrated cognitive impairment on the Mini-Mental State Examination (MMSE <23); combining subjective complaints with intact MMSE scores (≥23) allowed identification of 37% of the total sample as meeting minimum criteria for SCD.^18^

Exclusion criteria included studies focusing exclusively on specific disease populations (eg, stroke, HIV, Parkinson's disease) unless general population data were separately reported, case reports, reviews, editorials, commentaries, studies lacking sufficient diagnostic detail or original prevalence data, and non-human studies. When data were incomplete, authors were contacted to request additional prevalence estimates or methodological details.

### Data Extraction and Harmonization

Two reviewers independently extracted data using a standardized template, with a third reviewer crossvalidating 20% of extractions. Extracted variables included study characteristics (country, region, design, setting), participant demographics (sample size, mean age, sex distribution), methodological details (diagnostic criteria, assessment instruments, dementia exclusion), and prevalence outcomes. Demographics were harmonized across studies (age, sex, education, marital status), with education categorized as Low/Medium/High and marital status as Married/Not Married. Cardiometabolic and lifestyle risk factors, were extracted and harmonized into binary variables. The prevalence of MCI was calculated as the number of cases per screened population, and missing data were recorded as not reported (NR) without imputation. Duplicate datasets were resolved by retaining the most complete or informative source. Heterogeneity in diagnostic criteria, study setting, and sampling approach was acknowledged and explored through subgroup analyses, but was not statistically adjusted in prevalence calculations.

### Statistical Analysis

Pooled prevalence estimates were calculated using a random-effects meta-analysis with the DerSimonian–Laird estimator to account for between-study variability. Within-study variance was derived from the binomial sampling variance of individual prevalence estimates, while among-study variance was modelled through estimation of τ^2^; both variance components contributed to inverse-variance weighting in the pooled analyses. Heterogeneity was assessed using Higgins’ I^2^ statistic (with I^2^ ≥75% indicating substantial heterogeneity) and Cochran's Q-test (*P*<.10 considered significant). Between-study variance (τ^2^) was also estimated.

A random-effects model was prespecified given the anticipated clinical, methodological, and contextual heterogeneity across population-based studies conducted in diverse African settings; in instances of low heterogeneity, pooled estimates from the random-effects model approximate those obtained under a fixed-effect framework.

Sensitivity analyses were performed by restricting the meta-analysis to subsets of studies, and potential publication bias was evaluated through visual inspection of funnel plots. All analyses were implemented in R (version 4.5.0; R Foundation for Statistical Computing, Vienna, Austria). Subgroup analyses examined variability by geographical region, diagnostic criteria, assessment instrument, study setting, age group, and sex.

Meta-regression was conducted to identify demographic and cardiometabolic predictors of MCI prevalence. Meta-regression analyses were performed using univariable models due to the limited number of studies reporting complete covariate data, in order to minimise model overfitting and ensure stability of effect estimates. Publication bias was assessed using funnel plots and Egger's regression test, with *P*<.05 indicating significant asymmetry. Sensitivity analyses excluded studies with high risk of bias to evaluate robustness of pooled estimates.

### Quality Assessment

Methodological quality of included studies was independently assessed using the Joanna Briggs Institute (JBI) Critical Appraisal Checklist for Prevalence Studies.^19^ This tool evaluates representativeness, reliability, validity of diagnostic methods, and appropriateness of statistical analyses. Discrepancies were resolved by consensus or consultation with a third reviewer. Studies were classified as low, moderate, or high risk of bias.

### Ethical Considerations and Consent to Participate

Ethical approval and informed consent were not required for this study because it was based exclusively on secondary analysis of published, aggregate-level data, with no collection of individual participant data.

## RESULTS

### Study Characteristics

**FIGURE 1:**
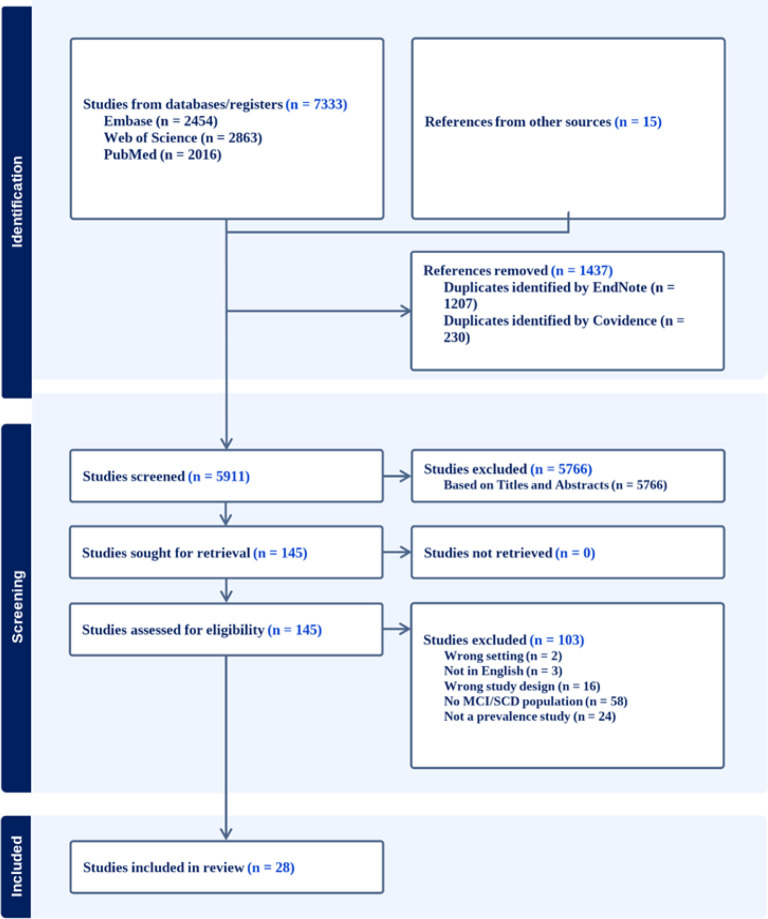
PRISMA flow chart

Twenty-eight studies addressing pre-dementia syndromes (SCD and MCI) from nine African countries were included (Figure 1); these are summarized in Table S4. Because several reports presented results for multiple countries (n=3) or for both SCD and MCI within a single sample (n=1), findings are displayed in dataset in Table S4. Studies were concentrated in West Africa (n=11; primarily Nigeria and Ghana),^20–30^ North Africa (n=6; all from Egypt),^31–36^ Central Africa (n=7; Republic of Congo and Central African Republic),^37–41^ East Africa (n=3; Ethiopia and Tanzania),^42–44^ and Southern Africa (n=2; South Africa).^18,45,46^ All studies were observational: the majority were cross-sectional (n=25) and four used longitudinal/cohort designs.^23,30,40,43^ Recruitment settings were predominantly community-based (n=28), with smaller numbers from residential/mixed settings (n=3) and clinical/hospital samples (n=1).

### Sample size and Case Counts

32 entries, 26,067 participants and 5,924 cases (SCD, 2,318 participants, 1,162 cases; MCI, 23,749 participants, and 4,762 cases). Reported prevalence varied widely: SCD, 44.6% to 53.5%^32,46^; MCI, 1.7% to 63.6%,^26,33^ reflecting heterogeneity in case definitions (eg, Petersen's criteria, DSM-V), instruments (MoCA, MMSE, CSI-D), sampling frames and settings.

### Sociodemographic and Clinical

In studies of MCI, participant characteristics varied widely (Table S5). Reported ranges included: marital status (17.9% to 94.0%),^40,44^ low education (4.0% to 98.1%),^24,27^ hypertension (prevalence>50% in several cohorts),^37,45^ diabetes (0% to 64.2%),^27,36^ smoking (0.5% to 74.1%),^31,44^ and family history (8.0% to 45.8%, though reported inconsistently).^36,42^

Overall, reporting of participant characteristics was also inconsistent in SCD studies (Table S6). Education was the most consistently described factor, ranging from very low (mean 2.1 years)^39^ to moderate (mean 9 to 12 years)^32,46^ across cohorts. In contrast, only some studies provided information on marital status or family history.

### Meta-Analysis of Prevalence

**FIGURE 2:**
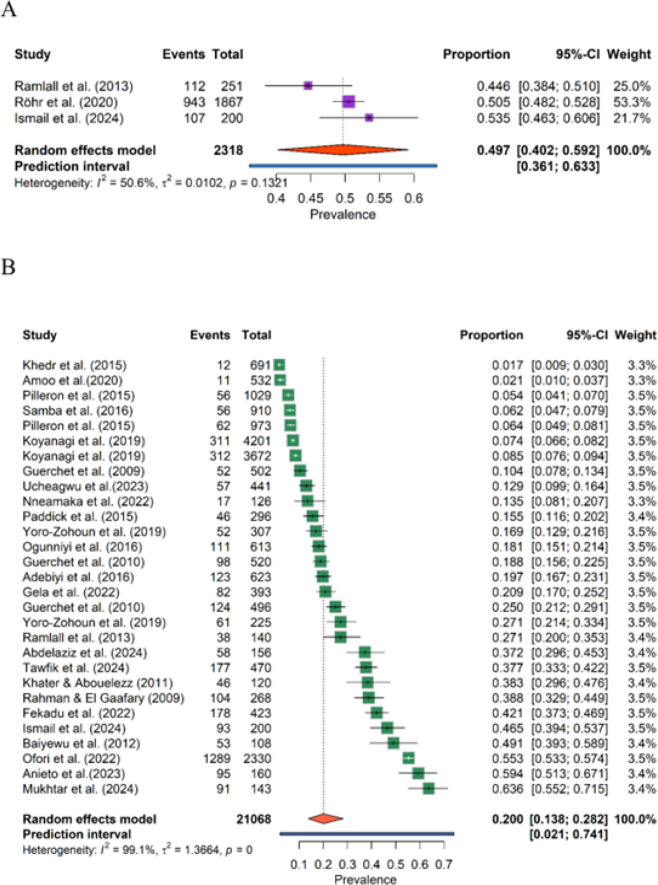
Forest Plot of the Meta-Analysis of Cognitive Impairment Prevalence in Africa

**FIGURE S1:**
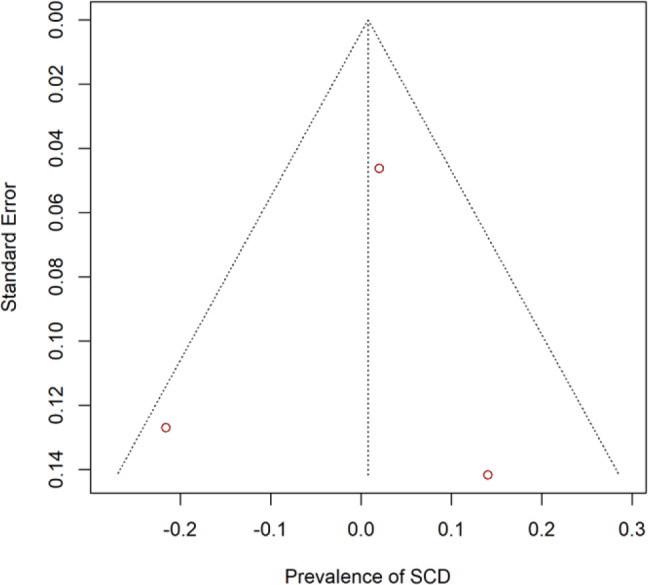
Funnel Plot for Publication Bias Assessments for SCD Studies

**FIGURE S2:**
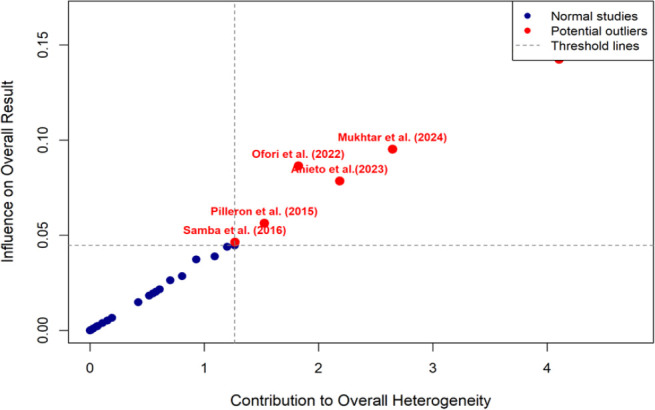
Baujat Plot-Study Influence and Heterogeneity Contribution

**FIGURE S3:**
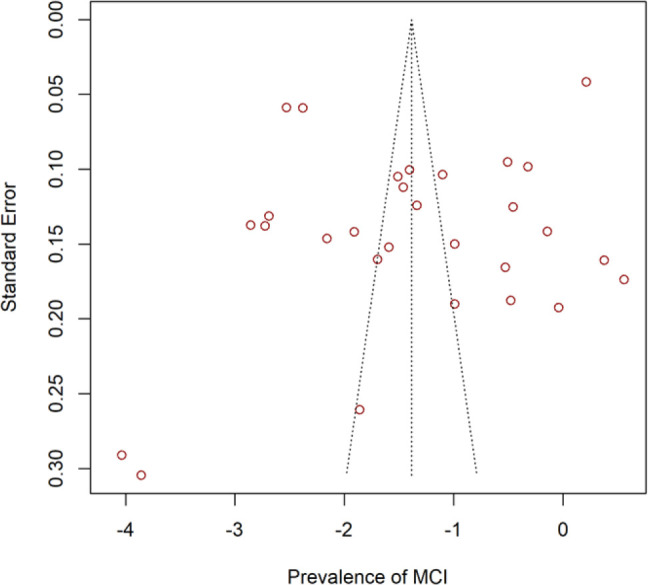
Funnel Plot for Publication Bias Assessments for MCI Studies

Using a random-effects approach, the pooled prevalence of SCD was 49.7% (95% CI: 40.1 to 59.2%), with low heterogeneity among the SCD studies (I^2^ 27.0%, τ^2^=.0018) (Figure 2A and Table S7). A sensitivity analysis, restricted to the three SCD studies, produced a similar pooled estimate of 49.7% (95% CI, 40.1 to 59.2%), with I^2^=50.6% (Table S8). A funnel plot analysis did not show significant asymmetry, suggesting a low likelihood of publication bias influencing the results (Figure S1). By contrast, the pooled prevalence of MCI across the included studies was 20.0% (95% CI, 14.0% to 27.9%), with extreme heterogeneity observed (I^2^=99.1%, τ^2^=1.3984) (Figure 2B and Table S9). Sensitivity analysis showed little change in the pooled prevalence across different scenarios, with the estimate remaining stable at 20.0% (95% CI, 13.8% to 28.2%) when excluding the smallest or largest study (Table S10). The Baujat plots highlight a small number of studies that contribute disproportionately to heterogeneity (Figure S2), but sensitivity analyses excluding these studies did not materially alter the pooled point estimate. Egger's regression did not indicate significant small-study/publication bias (*P*=.2949), suggesting that the observed heterogeneity is unlikely to be due to bias in smaller studies. Furthermore, the funnel plot analysis showed no clear asymmetry (Figure S3).

(A) Studies reporting subjective cognitive decline (SCD). (B) Studies reporting mild cognitive impairment (MCI)

### Subgroup Analyses

Pre-specified subgroup analyses were performed to explore sources of heterogeneity (Table 1).

**TABLE 1:** Subgroup Analysis of Mild Cognitive Impairment Prevalence by Demographic, Geographic, and Methodological Factors

Subgroup Analyses	No. of Studies	Prevalence, 95% CI (%)	12 (%)	*P* Values Within Subgroups	*P* Values Across Subgroups
Age (years)					
60–64	3	10.98 (6.88–17.08)	97.4	<.001	.1700
65–69	9	27.10 (17.19–39.95)	97.4	<.001	
70–74	10	14.73 (8.67–23.90)	98.2	<.001	
75–79	3	23.24 (16.75–31.29)	79.5	<.001	
80–84	3	21.39 (7.73–46.93)	97.5	.0304	
Region					
Central Africa	7	12.85 (7.49–21.18)	97.4	<.001	.6700
East Africa	3	24.81 (12.40–43.47)	97.2	.0102	
North Africa	6	27.33 (16.34–42.01)	96.7	.0035	
Southern Africa	2	15.53 (4.50–41.76)	97.9	.0147	
West Africa	11	21.84 (10.63–39.62)	99.5	.0034	
Diagnostic criteria					<.001
MCI / pAD Criteria	12	11.08 (8.00–15.14)	96.9	<.001	
Neuropsych Cutoffs	10	31.33 (21.52–43.15)	97.0	.0025	
Study Criteria	5	20.74 (12.79–31.82)	95.9	<.001	
Design					.7039
Cohort	4	16.84 (6.90–35.62)	97.7	.0018	
Cross-sectional	25	20.57 (13.91–29.34)	99.2	<.001	
Setting					.4433
Mixed	14	15.83 (8.31–28.08)	99.5	<.001	
Rural	3	14.89 (9.96–21.67)	89.0	<.001	
Urban	11	25.76 (18.50–34.66)	96.0	<.001	
Population source					.1566
Community	23	16.14 (10.41–24.16)	99.3	<.001	
Community & clinic	2	41.65 (33.35–50.45)	78.0	.0628	
Residential Care	3	34.16 (27.56–41.43)	56.4	<.001	
Sampling					.5254
Convenience	9	23.64 (17.48–31.13)	95.1	<.001	
Probability	20	18.49 (11.11–29.16)	99.4	<.001	
Subtype					.8345
Amnestic MCI	4	6.67 (2.22–18.38)	96.8	<.001	
Non-Amnestic MCI	3	5.76 (2.50–12.71)	90.3	<.001	
Survey start time					.0547
2001–2010	8	18.48 (11.38–28.59)	98.5	<.001	
2011–2020	13	14.73 (8.77–23.68)	98.4	<.001	
2021–2022	2	23.57 (7.71–53.26)	94.6	.0777	
NR	6	37.48 (23.97–53.28)	98.0	.1189	

Abbreviations: MCI – Mild Cognitive Impairment; pAD – prodromal Alzheimer's Disease; AD – Alzheimer's Disease; NR – Not Reported

**Age.** Prevalence varied across age bands, but heterogeneity remained high within strata (eg, 60 to 64 years: 10.98%, 95% CI, 6.88% to 17.08%, I^2^, 97.4%; 65 to 69 years: 27.10%, 95% CI, 17.19% to 39.95%, I^2^, 97.4%). The subgroup test across age categories was not statistically significant (*P* =.1700).

**FIGURE 3:**
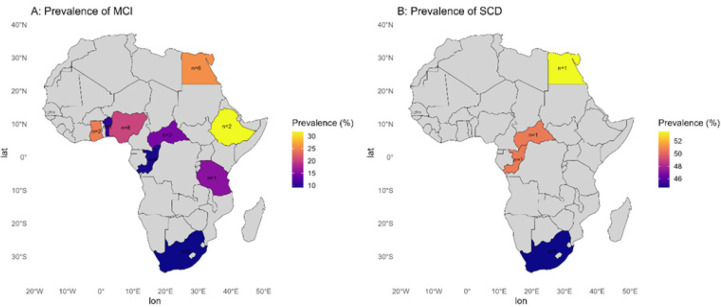
Geographic Distribution of Cognitive Impairment Prevalence in Africa, Based on Subgroup Analyses

**Country and region.** Country-level estimates varied numerically (Ethiopia: 30.50%; Egypt: 27.33%; Nigeria: 23.38%; Ghana: 23.94%; lower estimates in Central African Republic, South Africa, and Congo); however, these differences were not statistically significant (Figure 3, Table S11). Within-country heterogeneity remained substantial.). Regionally, North Africa had the highest pooled prevalence (27.33%) and Central Africa the lowest (12.85%); however, these regional differences were not statistically significant). Heterogeneity across regions was high.

(A) Prevalence of mild cognitive impairment (MCI) across different African regions. (B) Prevalence of subjective cognitive decline (SCD) across different African regions. Color intensity corresponds to the reported prevalence in each region, highlighting geographic variation.

**Diagnostic criteria.** Diagnostic approach explained a substantial portion of between-study variation (*P*<.001). Studies using neuropsychological test cut-offs reported the highest pooled prevalence of 31.33%; 95% CI, 21.52% to 43.15%; I^2^=97.0%), study-specific criteria produced an intermediate estimate of 20.74%; 95% CI 12.79% to 31.82%; I^2^=95.9%), and formal MCI/prodromal-AD research criteria yielded the lowest pooled prevalence of 11.08%; 95% CI 8.00% to15.14%; I^2^=96.9%).

**Design and setting.** Cross-sectional and cohort studies yielded similar pooled estimates (20.57% and 16.84% respectively; *P* across designs=.7039). Urban samples showed a numerically higher pooled prevalence (25.76%) than mixed (15.83%) or rural (14.89%) samples; however, these differences were not statistically significant).

**Population source and sampling.** Community-and-clinic samples and residential care samples showed higher prevalences (41.65% and 34.16%, respectively) than community samples alone (16.14%); convenient and probability sampling produced comparable pooled prevalences (*P* across sampling methods=.5254).

**MCI subtype and survey timing.** Reported amnestic and non-amnestic prevalences were similar and low (≈6% each), but these estimates are based on very few studies (4 and 3 studies, respectively). Studies with unspecified survey start dates had higher pooled prevalences than those with documented start periods.

Across nearly all subgroup comparisons, within-group heterogeneity remained substantial, indicating that subgroup stratification explained only a fraction of the total variance.

### Meta-Regression

**FIGURE 4:**
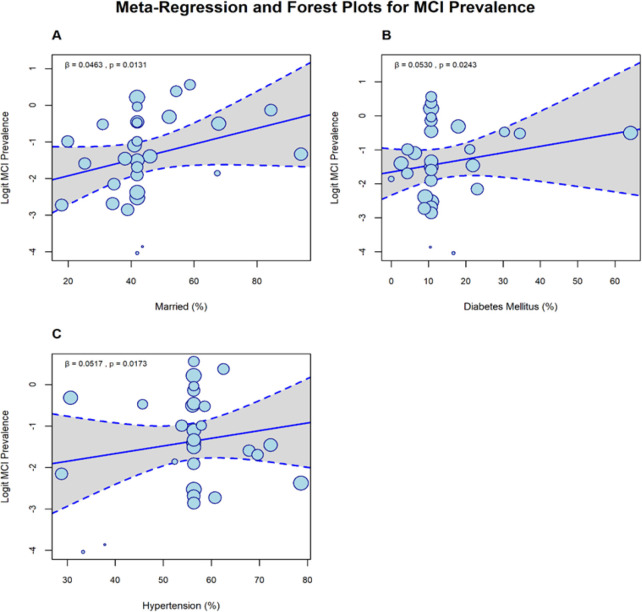
Meta-Regression of Mild Cognitive Impairment (MCI) Prevalence Showing Significant Predictors.

A multivariable random-effects meta-regression (29 studies data set) examined study-level predictors of reported MCI prevalence (Table S11). The model intercept was not significant (β=−4.3267, SE=2.9032, *P*=.1361). Independent positive predictors were percentage married (β=0.0463, SE = 0.0187; 95% CI 0.0097 to 0.0829, *P*=.0131); diabetes prevalence (β = 0.0530, SE = 0.0235; 95% CI 0.0069 to 0.0991, *P*=.0243); and hypertension prevalence (β = 0.0517, SE = 0.0217; 95% CI 0.0091 to 0.0942, *P*=.0173) Figure 4. The 60–64 age indicator was negatively associated with reported prevalence (β=−3.2169, SE = 1.0719; *P*=.0027); education, smoking, family history and most sex/age strata were nonsignificant. The model explained ∼53.4% of between-study variance (R^2^ = 53.4%), and VIFs (range 1.44 to 2.87; Table S12) indicate no serious multicollinearity.

(A) Married (%), (B) Diabetes mellitus (%), (C) Hypertension (%). Bubble size reflects study weight; trend lines indicate the direction of association with MCI prevalence.

### Quality Assessment of Included Studies

Of the 28 included studies, 26 were of high methodological quality (≥70% of criteria rated “Yes”), indicating adequate sampling, validated measurement tools, and appropriate statistical analyses, while two studies (Ofori et al, and Fekadu et al),^28,42^ were rated as of moderate quality due to reliance on self-reported data and unclear response rates (Table S13).

## DISCUSSION

This systematic review and meta-analysis provide the first comprehensive estimates of pre-dementia prevalence in Africa, a region largely absent from global data. SCD was strikingly common, affecting nearly half of older adults, while MCI affected 20.0% (95% CI 14.0 to 27.9%). The MCI estimate, however, was constrained by extreme heterogeneity (I^2^≈99%), reflecting methodological rather than sampling differences.

Use of neuropsychological cut-offs alone produced prevalence nearly three times higher than clinical criteria, exposing a critical lack of diagnostic standardization. Meta-regression further linked MCI prevalence to cardiometabolic risk factors, particularly hypertension and diabetes, highlighting modifiable contributors to cognitive decline. Collectively, these findings redefine the regional and global narrative on cognitive ageing and establish priorities for harmonized diagnosis, research, and public health interventions.

### Comparison with Global Evidence

Our pooled MCI prevalence of 20.0% exceeds the global average of 15.56% reported by Bai et al,^9^ but aligns closely with the 23.7% estimate from Salari et al.^11^ Earlier global reviews incorporated very few African studies (4–8 per review) and could not conduct meaningful regional analyses.^9,11^ By synthesizing 28 studies from nine countries, including African-specific sources, our review provides the most representative continental estimate to date.

Prior global reviews reported higher African prevalence (eg, 26.4% in Salari et al),^11^ likely reflecting methodological artefacts rather than true epidemiological differences. Subgroup analyses demonstrate that studies applying established clinical criteria (eg, Petersen's, NIA-AA) yielded a more conservative prevalence of 11.08%, consistent with global averages.^9^ This underscores the urgent need for standardization of diagnostic criteria and culturally adapted assessment tools, addressing concerns raised by McGrattan et al.^10^ regarding inconsistent methodologies in LMICs.

### Clinical and Public Health Implications

The high prevalence of SCD identifies a substantial population of older adults aware of cognitive changes but not yet meeting MCI criteria.^4^ This group represents an important target for low-cost, community-based interventions aimed at mitigating modifiable risk factors.^47,48^ The observed associations between MCI prevalence and hypertension and diabetes strengthen the case for integrating cognitive health screening into routine primary care management of cardiometabolic diseases.^49–51^ Such strategies are feasible, scalable, and likely to reduce the dual burden of dementia and cardiovascular disease in African populations.^51^

The contrast in prevalence between community-based and clinical/residential settings highlights unmet needs in the community, emphasizing the importance of outreach and engagement to identify at-risk individuals before progression to more severe cognitive impairment.^52,53^ Given the variability in MCI prevalence by diagnostic approach, incorporating objective functional measures – such as gait assessment – may enhance early detection.^54^ Dual-task gait paradigms, particularly those incorporating rhythmic auditory cueing (RAC), which engage motor–cognitive coupling, could serve as sensitive probes of subtle pre-dementia changes warranting validation in large population studies.^55,56^

### Strengths

This review's principal strength is its comprehensiveness, encompassing a larger and more diverse African sample than prior studies. The inclusion of 28 studies from multiple sources and the application of metaanalytic techniques—including subgroup analyses and meta-regression—enabled systematic exploration of heterogeneity. Additionally, harmonization of demographic, educational, and cardiometabolic variables across studies improved comparability and allowed identification of potential risk factors for MCI and SCD.

### Limitations

Limitations include the predominance of cross-sectional designs, which preclude causal inference and assessment of progression from SCD or MCI to dementia. Rural populations were underrepresented, despite being the majority demographic in Africa. Variability in diagnostic thresholds, limited use of biomarker validation, and sparse longitudinal follow-up restrict prognostic interpretation. Sparse longitudinal follow-up further limits assessment of progression. Finally, crude categorizations of education and lifestyle factors may have masked their role as potential predictors.

## CONCLUSION

Pre-dementia states are highly prevalent across Africa: SCD affects nearly half of older adults, and MCI affects one in five. However, prevalence estimates are heavily influenced by methodological choices, particularly diagnostic criteria. Standardized, culturally validated approaches that incorporate clinical judgment and functional assessment beyond neuropsychological cut-offs are urgently needed.

Longitudinal, population-representative studies are essential to clarify MCI incidence and conversion to dementia. The strong association with cardiometabolic comorbidities presents a clear opportunity for integrated public health strategies, where effective management of hypertension and diabetes could reduce both cognitive decline and cardiovascular disease.

By assembling the largest and most representative African dataset to date, this review fills a critical knowledge gap and provides a robust foundation for research standardization, evidence-based policy, and the design of effective dementia prevention and care strategies across the continent.

## Figures and Tables

Key to abbreviation: SDC, subjective cognitive decline.

Key to abbreviation: MCI, mild cognitive impairment

## Appendix

**TABLE S1:** PubMed Search Strategy Including Full Search Terms (March 2025)

Line	Keywords	Records Retrieved
#1 Cognitive impairment	((“subjective memory complaint*”[Title/Abstract] OR “subjective memory impairment”[Title/Abstract] OR “subjective cognitive complaint*”[Title/Abstract] OR “subjective cognitive impairment” [Title/Abstract] OR “subjective cognitive decline”[Title/Abstract] OR “memory complaint*”[Title/Abstract] OR “cognitive complaint*” [Title/Abstract] OR “subjective cognitive”[Title/Abstract] OR “subjective memory”[Title/Abstract] OR “SCD”[Title/Abstract] OR “subtle cognitive decline”[Title/Abstract] OR “early diagnosis” [Title/Abstract] OR “Stage”[All Fields]) AND “preclinical” [Title/Abstract]) OR “asymptomatic”[Title/Abstract] OR “pre-MCI” [Title/Abstract] OR “preMCI”[Title/Abstract] OR “pre-MCI” [Title/Abstract] OR “dementia”[All Fields] OR “mild cognitive impairment”[Title/Abstract] OR “mild neurocognitive disorder” [Title/Abstract] OR “neurocognitive disorder”[Title/Abstract] OR “cogniti*”[Title/Abstract] OR “neurocognitive disorders”[MeSH Terms] OR “Alzheimer's Disease” [Title/Abstract]	1,093,254
#2 Age (Older adults)	“old”[Title/Abstract] OR “older adults”[Title/Abstract] OR “senior” [Title/Abstract] OR “elder*”[Title/Abstract] OR “aged”[Title/Abstract] OR “aged”[MeSH Terms]	5,420,195
#3 Prevalence	“incidence”[Title/Abstract] OR “prevalence”[Title/Abstract] OR “occurrence”[Title/Abstract] OR “rate*”[Title/Abstract] OR “ epidemiolog*”[Title/Abstract] OR “general population”[Title/Abstract] OR “community”[Title/Abstract] OR “population”[Title/Abstract]	7,462,111
#4 Africa	Africa[Title/Abstract] OR subSaharan Africa[Title/Abstract] OR Algeria [Title/Abstract] OR Egypt[Title/Abstract] OR Libya[Title/Abstract] OR. Morocco[Title/Abstract] OR Tunisia[Title/Abstract] OR Cameroon [Title/Abstract] OR Central African Republic[Title/Abstract] OR Chad [Title/Abstract] OR Congo[Title/Abstract] OR Democratic Republic of the Congo[Title/Abstract] OR Equatorial Guinea[Title/Abstract] OR Gabon[Title/Abstract] OR Burundi[Title/Abstract] OR Djibouti [Title/Abstract] OR Eritrea[Title/Abstract] OR Ethiopia[Title/Abstract] OR Kenya[Title/Abstract] OR Rwanda[Title/Abstract] OR Somalia [Title/Abstract] OR Sudan[Title/Abstract] OR South Sudan[Title/Abstract] OR Tanzania[Title/Abstract] OR Uganda[Title/Abstract] OR Angola[Title/Abstract] OR Botswana[Title/Abstract] OR Lesotho[Title/Abstract] OR Mozambique[Title/Abstract] OR Ivory Coast[Title/Abstract] OR Namibia[Title/Abstract] OR South Africa[Title/Abstract] OR Swaziland [Title/Abstract] OR Zambia[Title/Abstract] OR Zimbabwe[Title/Abstract] OR Benin[Title/Abstract] OR Burkina Faso[Title/Abstract] OR Côte d’Ivoire [Title/Abstract] OR Gambia[Title/Abstract] OR Ghana[Title/Abstract] OR Guinea[Title/Abstract] OR Guinea-Bissau[Title/Abstract] OR Liberia [Title/Abstract] OR Mali[Title/Abstract] OR Malawi[Title/Abstract] OR Mauritania[Title/Abstract] OR Mauritius[Title/Abstract] OR Eswatini [Title/Abstract] OR Madagascar[Title/Abstract] OR Niger[Title/Abstract] OR Nigeria[Title/Abstract] OR Senegal[Title/Abstract] OR Sierra Leone [Title/Abstract] OR Togo[Title/Abstract]	540,174
#5	(((#1) AND (#2)) AND (#3)) AND (#4)	2,016

Abbreviations: SCD, Subjective Cognitive Decline; MCI, Mild Cognitive Impairment; pre-MCI, Pre–Mild Cognitive Impairment; AD, Alzheimer's Disease.

**TABLE S2:** Web of Science Search Strategy, Including Full Search Terms (2025 March)

Line	Keywords	Records Retrieved
#1 Cognitive impairment	TS=((“subjective memory complaint*” OR “subjective memory impairment” OR “subjective cognitive complaint*” OR “subjective cognitive impairment” OR “subjective cognitive decline” OR “memory complaint*” OR “cognitive complaint*” OR “subjective cognitive” OR “subjective memory” OR “SCD” OR “subtle cognitive decline” OR “early diagnosis” OR “Stage” AND “preclinical” OR “asymptomatic” OR “pre-MCI” OR “preMCI” OR “pre-MCI” OR “dementia” OR “mild cognitive impairment” OR “mild neurocognitive disorder” OR “neurocognitive disorder” OR “cogniti*” OR “Alzheimer's Disease”))	1,890,465
#2 Age (Older adults)	TS=(old OR older adults OR senior OR elder* OR aged)	12,261,111
#3 Prevalence	TS=(“incidence” prevalence OR occurrence OR rate* OR epidemiolog* OR “general population” OR community OR population)	15,484,712
#4 Africa	TS=(Africa OR subSaharan Africa OR Algeria OR Egypt OR Libya OR Morocco OR Tunisia OR Cameroon OR Central African Republic OR Chad OR Congo OR Democratic Republic of the Congo OR Equatorial Guinea OR Gabon OR Burundi OR Djibouti OR Eritrea OR Ethiopia OR Kenya OR Rwanda OR Somalia OR Sudan OR South Sudan OR Tanzania OR Uganda OR Angola OR Botswana OR Lesotho OR Mozambique OR Ivory Coast OR Namibia OR South Africa OR Swaziland OR Zambia OR Zimbabwe OR Benin OR Burkina Faso OR Coôte d’Ivoire OR Gambia OR Ghana OR Guinea OR Guinea-Bissau OR Liberia OR Mali OR Malawi OR Mauritania OR Mauritius OR Eswatini OR Madagascar OR Niger OR Nigeria OR Senegal OR Sierra Leone OR Togo)	1,301,743
#5	#1 AND #2 AND #3 AND #4	9,281
#6 Animal studies	TS=((rat OR rats OR animal OR animals OR mice OR “in vivo” OR mouse OR rabbit OR rabbits OR murine OR pig OR pigs OR dog OR dogs OR bovine OR fish OR vertebrate OR vertebrates OR cat OR cats OR rodent OR rodents OR mammal OR mammals OR chicken OR chickens OR monkey OR monkeys OR sheep OR canine OR canines OR porcine OR cattle OR bird OR birds OR hamster OR hamsters OR primate OR primates OR cow OR cows OR chick OR horse OR horses OR avian OR avians OR calf OR swine OR swines OR xenopus OR turkeys OR bear OR bears OR frog OR frogs OR zebrafish OR goat OR goats OR equine OR calves OR poultry OR macaque OR macaques OR mole OR moles OR ovine OR lamb OR lambs OR fishes OR diptera OR amphibian OR amphibians OR snake OR snakes OR ruminant OR ruminants OR hen OR hens OR piglet OR piglets OR feline OR felines OR simian OR simians OR laevis OR trout OR trouts OR teleost OR teleosts OR salmon OR salmons OR seal OR seals OR bull OR bulls OR ewe OR ewes OR hedgehog OR hedgehogs OR macaca OR macacas OR proteus OR pigeon OR pigeons OR bat OR bats OR duck OR ducks . OR chimpanzee OR chimpanzees OR baboon OR baboons OR deer OR rana OR ranas OR carp OR carps OR heifer OR swallow OR swallows OR lizard OR lizards OR canis OR sow OR sows OR cynomolgus OR quail OR quails OR reptile OR reptiles OR turtle OR turtles OR buffalo OR gerbil OR gerbils OR boar OR boars OR squirrel OR squirrels OR oncorhynchus OR mus OR toad OR toads OR fowl OR fowls OR rerio OR danio OR ara OR aras OR musculus OR tadpole OR tadpoles OR mulatta OR salmo OR ram OR eagle OR, eagles OR ferret OR ferrets OR goldfish OR catfish OR whale OR whales OR fox OR foxes OR ape OR apes OR elephant OR elephants OR bos OR marmoset OR marmosets OR cod OR cods OR shark OR sharks OR wolf OR eel OR eels OR auratus OR rattus OR zebra OR zebras OR tilapia OR tilapias OR gilt OR camel OR camels OR squid OR gallus OR marsupial OR marsupials OR vole OR voles OR fascicularis OR ovis OR salmonid OR salmonids OR tiger OR tigers OR dolphin OR dolphins OR robin OR robins OR carpio OR opossum OR opossums OR cyprinus OR salamander OR salamanders OR felis OR mink OR minks OR swan OR swans OR norvegicus OR bufo OR torpedo OR bass OR lamprey OR lampreys OR sus OR python OR pythons OR tetrapod OR tetrapods OR shrew OR shrews OR lion OR lions OR hog OR hogs OR songbird OR songbirds OR oreochromis OR starling OR starlings OR caprine OR carassius OR owl OR owls OR newt OR newts OR papio OR scrofa OR hare OR hares OR gorilla OR gorillas OR flounder OR flounders OR goose OR herring OR herrings OR therian OR buffaloes OR canary OR sparrow OR sparrows OR microtus OR octopus OR troglodytes OR tuna OR amphibia OR chinchilla OR chinchillas OR ide OR oryzias OR cervus OR kangaroo OR kangaroos OR armadillo OR armadillos OR callithrix OR “pan troglodytes” OR saimiri OR cichlid OR cichlids OR donkey OR donkeys OR bream OR char OR chars OR finch OR raccoon OR . raccoons OR bothrops OR anguilla OR perch OR cricetus OR seabird OR seabirds OR buck OR bucks OR naja OR coturnix OR salmonids OR geese OR minnow OR minnows OR raptor OR raptors OR merione OR meriones OR rodentia OR elaphus OR amniote OR amniotes OR elasmobranch OR emu OR emus OR peromyscus OR hominid OR hominids OR bubalus OR crotalus OR gull OR gulls OR anas OR anura OR lemur OR lemurs OR crow OR crows OR camelus OR gibbon OR gibbons OR waterfowl OR parrot OR parrots OR eels OR cob OR stickleback OR sticklebacks OR columba OR mesocricetus OR ambystoma OR raven OR ravens OR gadus OR penguin OR penguins OR orangutan OR orangutans OR sturgeon OR sturgeons OR cuniculus OR aves OR virginianus OR cephalopod OR cephalopods OR cebus OR sparus OR tortoise OR tortoises OR guttata OR morhua OR unguiculatus OR dogfish OR vulpes OR mallard OR mallards OR apodemus OR alligator OR alligators OR oryctolagus OR llama OR llamas OR reindeer OR mustela OR duckling OR ducklings OR wolves OR sander OR amazona OR zebu OR badger OR badgers OR dove OR doves OR ictalurus OR capra OR capras OR equus OR camelid OR camelids OR poecilia OR mule OR mules OR perciformes OR salvelinus OR labrax OR cyprinidae OR ariidae OR crocodile OR crocodiles OR fundulus OR dicentrarchus OR clarias OR cercopithecus OR chiroptera OR alpaca OR alpacas OR pike OR pikes OR paralichthys OR puma OR pumas OR didelphis OR pisces OR macropus OR triturus OR bison OR bisons OR epinephelus OR gasterosteus OR panthera OR acipenser OR mackerel OR mackerels OR tamarin OR tamarins OR ostrich OR anolis OR vervet OR vervets OR wallaby OR glareolus OR beaver OR beavers OR dromedary OR catus OR killifish OR pimephales OR promelas OR aotus OR phoca OR panda OR pandas OR porpoise OR porpoises OR myotis OR yak OR yaks OR agkistrodon OR vipera OR otter OR otters OR turbot OR turbots OR squamate OR carnivora OR mullet OR, mullets OR hawk OR hawks OR taeniopygia OR seahorse OR seahorses OR “poecilia reticulata” OR falcon OR falcons OR prosimian OR prosimians OR parus OR perca OR fingerling OR fingerlings OR antelope OR antelopes OR tupaia OR passeriformes OR sepia OR saguinus OR coyote OR coyotes OR pongo OR meleagris OR reptilia OR lepus OR psittacine OR hagfish OR warbler OR warblers OR “russell s viper” OR “russell s vipers” OR smolt OR smolts OR budgerigar OR sardine OR sardines OR cavia OR cavias OR hyla OR pleurodeles OR siluriformes OR “great tit” OR “great tits” OR guppy OR bonobo OR bonobos OR rutilus OR trichosurus OR muridae OR phodopus OR channa OR squalus OR lynx OR sturnus OR petromyzon OR vitulina OR monodelphis OR cuttlefish OR adder OR adders OR, lepomis OR canaria OR gambusia OR guppies OR xiphophorus OR flatfish OR koala OR koalas OR labeo OR stingray OR stingrays OR chelonia OR lampetra OR spermophilus OR crocodilian OR “passer domesticus” OR sciurus OR artiodactyla OR ranidae OR corvus OR, necturus OR platypus OR canaries OR bovid OR lagopus OR trimeresurus OR gariepinus OR marten OR martens OR drosophilidae OR mugil OR sunfish OR porcellus OR cypriniformes OR alouatta OR scophthalmus OR anser OR electrophorus OR putorius OR iguana OR iguanas OR lama OR lamas OR takifugu OR circus OR eptesicus OR flycatcher OR galago OR galagos OR trachemys OR lungfish OR characiformes OR shorebird OR shorebirds OR giraffe OR giraffes OR micropterus OR scyliorhinus OR cichlidae OR loligo OR porcupine OR porcupines OR chub OR chubs OR solea OR pleuronectes OR hylidae OR viperidae OR echis OR sorex OR anchovy OR lagomorph OR ostriches OR vulture OR vultures OR whitefish OR araneus OR jird OR jirds OR tern OR esox OR drake OR drakes OR elapidae OR gallopavo OR chordata OR myodes OR caretta OR serinus OR grouse OR misgurnus OR meles OR blackbird OR blackbirds OR coregonus OR bobwhite OR bobwhites OR heteropneustes OR mammoth OR mammoths OR turdus OR rhinella OR ateles OR characidae OR clupea OR bungarus OR brill OR “struthio camelus” OR sloth OR sloths OR pteropus OR sculpin OR anthropoids OR pollock OR pollocks OR morone OR “pan paniscus” OR litoria OR chipmunk OR chipmunks OR balaenoptera OR marmota OR melopsittacus OR hyrax OR lemming OR lemmings OR halibut OR hylobates OR lates OR caiman OR caimans OR sigmodon OR stenella OR barbel OR barbels OR sterna OR parakeet OR parakeets OR phocoena OR leptodactylus OR canidae OR buteo OR harengus OR gopher OR gophers OR marmot OR marmots OR gosling OR goslings OR platichthys OR gar OR gars OR sebastes OR marsupialia OR notophthalmus OR gazelle OR gazelles OR insectivora OR paridae OR felidae OR russula OR galliformes OR bombina OR colobus OR echidna OR echidnas OR seabass OR syncerus OR plaice OR “blue tit” OR “blue tits” OR pagrus OR catfishes OR cetacea OR barbus OR cygnus OR ficedula OR chamois OR colubridae OR perches OR coelacanth OR fitch OR urodela OR cynops OR martes OR halichoerus OR aix OR salmonidae OR leuciscus OR magpie OR magpies OR silurus OR whiting OR whitings OR anseriformes OR colinus OR rhea OR chlorocebus OR octodon OR acinonyx OR mouflon OR mouflons OR ibex OR tetraodon OR bufonidae OR equidae OR jackal OR cephalopoda OR dendroaspis OR glama OR muskrat OR muskrats OR sable OR sables OR wildebeest OR streptopelia OR albifrons OR vespertilionidae OR woodpecker OR woodpeckers OR muntjac OR muntjacs OR archosaur OR branta OR cricetulus OR megalobrama OR poeciliidae OR desmodus OR snakehead OR snakeheads OR tench OR teal OR teals OR bandicoot OR bandicoots OR apteronotus OR phyllostomidae OR crocidura OR buzzard OR buzzards OR larimichthys OR cercocebus OR pipistrellus OR erithacus OR impala OR impalas OR rousettus OR haddock OR haddocks OR tinca OR ratite OR calidris OR cynoglossus OR hypophthalmichthys OR bullock OR bullocks OR dromedaries OR alectoris OR filly OR salamandra OR cingulata OR bitis OR grus OR ammodytes OR macaw OR macaws OR hypoleuca OR sapajus OR cyprinodontiformes OR hippopotamus OR pelophylax OR capybara OR capybaras OR weasel OR weasels OR cairina OR cynomys OR lutra OR cockatoo OR cockatoos OR lachesis OR lagomorpha OR rupicapra OR daboia OR “orang utan” OR “orang utans” OR platyrrhini OR charadriiformes OR micrurus OR psittaciformes OR spalax OR loris OR mustelidae OR sylvilagus OR vitticeps OR cockatiel OR mustelus OR cottus OR erythrocebus OR dipodomys OR platessa OR callicebus OR loricariidae OR catostomus OR cuneata OR cyanistes OR cyprinodon OR sigmodontinae OR elasmobranchii OR trichechus OR sauropsid OR xenarthra OR dormouse OR perissodactyla OR nautilus OR cirrhinus OR gulo OR gulos OR tragelaphus OR merula OR numida OR sciaenidae OR cerastes OR sciuridae OR gibbosus OR octopuses OR eland OR elands OR phyllomedusa OR pogona OR walrus OR agamidae OR leptodactylidae OR ridibundus OR leontopithecus OR anteater OR anteaters OR pelodiscus OR cebidae OR columbianus OR “pelteobagrus fulvidraco” OR hominoidea OR mandrillus OR “zonotrichia leucophrys” OR agama OR gobiocypris OR “bearded dragon” OR “bearded dragons” OR sarotherodon OR talpa OR discoglossus OR hagfishes OR sphenodon OR gudgeon OR amphiuma OR aythya OR tenrec OR tenrec OR hominidae OR risoria OR salamandridae OR camelidae OR columbiformes OR latimeria OR plover OR plovers OR afrotheria OR “falco sparverius” OR polecat OR polecats OR crotalinae OR salvadora OR tarsier OR lucioperca OR anchovies OR lungfishes OR terrapin OR “dromaius novaehollandiae” OR lateolabrax OR eigenmannia OR pelamis OR theropithecus OR murinae OR gander OR gymnotus OR pseudacris OR gymnophiona OR gymnotiformes OR laticauda OR falconiformes OR dugong OR dugongs OR pintail OR pintails OR rook OR rooks OR lasiurus OR catshark OR catsharks OR micropogonias OR “red junglefowl” OR paddlefish OR eutheria OR ophiophagus OR hollandicus OR nymphicus OR pimelodidae OR aepyceros OR cobitidae OR strigiformes OR cobitis OR dormice OR alytes OR calloselasma OR guanaco OR guanacos OR phasianidae OR “round goby” OR trichogaster OR catarrhini OR eelpout OR eelpouts OR galaxias OR gaur OR pungitius OR suslik OR susliks OR flatfishes OR percidae OR caprinae OR todarodes OR osmerus OR ameiurus OR anthropoidea OR “castor canadensis” OR pouting OR poutings OR tetraodontiformes OR arvicolinae OR siamang OR siamangs OR “castor fiber” OR nomascus OR “red knot” OR “red knots” OR syngnathidae OR iguanidae OR eretmochelys OR ursidae OR callimico OR columbidae OR microhylidae OR anaxyrus OR menidia OR pipistrelle OR greylag OR pipidae OR scandentia OR bowfin OR bowfins OR dendrobatidae OR zenaida OR bushbaby OR harrier OR harriers OR macropodidae OR pygerythrus OR clupeidae OR odorrana OR corvidae OR jerboa OR jerboas OR canutus OR hylobatidae OR clupeiformes OR “great cormorant” OR “great cormorants” OR scorpaeniformes OR chondrostean OR garfish OR proboscidea OR psetta OR diapsid OR serotinus OR tetrao OR walruses OR carcharhiniformes OR leucoraja OR pumpkinseed OR dosidicus OR acipenseriformes OR daubentonii OR emberizidae OR gadiformes OR hyraxes OR stizostedion OR wolverine OR wolverines OR lissotriton OR acanthurus OR centrarchidae OR gloydius OR laurasiatheria OR limosa OR psittacula OR leporidae OR proteidae OR zander OR zanders OR arapaima OR bagridae OR cyprinodontidae OR mithun OR pandion OR jackdaw OR jackdaws OR procyonidae OR carus OR jaculus OR salmoniformes OR “common sole” OR “common soles” OR protobothrops OR calamita OR brachyteles OR trionyx OR turdidae OR boidae OR luscinia OR pugnax OR euarchontoglires OR saithe OR saithes OR symphalangus OR aardvark OR aardvarks OR oystercatcher OR oystercatchers OR arius OR corydoras OR poacher OR poachers OR aurochs OR cebuella OR crecca OR lemuridae OR sirenia OR lemmus OR perdix OR glires OR lepidosaur OR muskox OR deinagkistrodon OR pholidota OR holocephali OR cercopithecinae OR clariidae OR agapornis OR doryteuthis OR tyrannidae OR dicroglossidae OR godwit OR godwits OR monedula OR pongidae OR atheriniformes OR colobinae OR lophocebus OR atelidae OR cottidae OR leucopsis OR acanthuridae OR didelphimorphia OR elver OR elvers OR lapponica OR dermoptera OR “european hake” OR “european hakes” OR gerbillinae OR banteng OR hartebeest OR hartebeests OR hogget OR haematopus OR “anguis fragilis” OR “grey heron” OR “grey herons” OR “blue whiting” OR “blue whitings” OR furnariidae OR macrovipera OR esocidae OR lapwing OR lapwings OR mylopharyngodon OR wallabia OR beloniformes OR potoroo OR potoroos OR “athene noctua” OR pleuronectidae OR bushbabies OR muscicapidae OR alligatoridae OR fuligula OR “bush baby” OR guineafowl OR spoonbill OR spoonbills OR viverridae OR catostomidae OR zebrafishes OR ibexes OR vendace OR estrildidae OR monotremata OR sepiella OR ambystomatidae OR shelduck OR shelducks OR treeshrew OR treeshrews OR hoplobatrachus OR pochard OR hoolock OR hoolocks OR lynxes OR antilope OR antilopes OR blackbuck OR blackbucks OR cricetinae OR paramisgurnus OR skylark OR skylarks OR soleidae OR allobates OR “northern wheatear” OR “northern wheatears” OR pitheciidae OR takin OR theria OR vanellus OR galaxiidae OR lorisidae OR ostralegus OR palaeognathae OR “stone loach” OR alauda OR callitrichinae OR caniformia OR duttaphrynus OR ictaluridae OR osteoglossiformes OR poultries OR curema OR “ruddy turnstone” OR “ruddy turnstones” OR sheatfish OR sunfishes OR centropomidae OR hemachatus OR platalea OR thamnophilidae OR “song thrush” OR atherinopsidae OR siluridae OR tadorna OR chroicocephalus OR ermine OR ermines OR gavialis OR ruffe OR tupaiidae OR diprotodontia OR hyaenidae OR antilopinae OR crocodylidae OR herpestidae OR hippopotamidae OR “northern shoveler” OR “round gobies” OR cheirogaleidae OR indriidae OR fundulidae OR pythonidae . OR rhynchocephalia OR anodorhynchus OR “red-backed shrike” OR “red-backed shrikes” OR triakidae OR phalangeridae OR aoudad OR boreoeutheria OR “eurasian jay” OR “eurasian jays” OR feliformia OR haplorhini OR osteoglossidae OR paenungulata OR struthioniformes OR ferina OR sanderling OR sanderlings OR spheniscidae OR cuttlefishes OR cygnet OR dasycneme OR gadwall OR gadwalls OR “pelobates fuscus” OR wryneck OR wrynecks OR afrosoricida OR culaea OR “dover sole” OR “dover soles” OR paralichthyidae OR passeridae OR osteolaemus OR “song thrushes” OR bluethroat OR bluethroats OR hydrophiidae OR megrim OR mephitidae OR strepsirhini OR tomistoma OR epidalea OR osmeriformes OR “bush babies” OR tarsiiform OR atelinae OR bufotes OR “eurasian coot” OR “eurasian coots” OR galagidae OR geopelia OR philomachus OR tubulidentata OR bombinatoridae OR pelobatidae OR tachysurus OR ailuridae OR woodlark OR woodlarks OR alcelaphinae OR redshank OR redshanks OR salientia OR “sand smelt” OR “sand smelts” OR woodmice OR woodmouse OR dasyproctidae OR “eurasian wigeon” OR “eurasian wigeons” OR garganey OR garganeys OR “lemon sole” OR “lemon soles” OR “common dab” OR “common dabs” OR graylag OR graylags OR leucorodia OR osphronemidae OR bewickii OR “common moorhen” OR “common moorhens” OR decapodiformes OR gobbler OR gobblers OR odontophoridae OR paddlefishes OR salmonine OR esociformes OR “eurasian woodcock” OR “eurasian woodcocks” OR “european smelt” OR “european smelts” OR goldfishes OR tenches OR tyranni OR “common chaffinch” OR “common chaffinchs” OR “common redstart” OR “common redstarts” OR “common roach” OR “common roachs” OR “great knot” OR “great knots” OR potoroidae OR alytidae OR coregonine OR dipteral OR leveret OR “poeciliopsis gracilis” OR amphiumidae OR . batrachoidiformes OR “bighead goby” OR heteropneustidae OR lullula OR “norway pout” OR “norway pouts” OR sipunculida OR dogfishes OR sebastidae OR tarsiidae OR alethinophidia OR “common nase” OR “common nases” OR “common sandpiper” OR “common sandpipers” OR “eurasian blackcap” OR “eurasian blackcaps” OR pterocnemia OR syngnathiformes OR “common chaffinches” OR eupleridae OR octopodiformes OR phascolarctidae OR scophthalmidae OR “starry smooth-hound” OR “starry smooth-hounds” OR whitefishes OR cuniculidae OR “european sprat” OR “ european sprats” OR “rosy bitterling” OR “rosy bitterlings” OR “common dace” OR “common daces” OR “lesser weever” OR “lesser weevers” OR scaldfish OR “water rail” OR “water rails” OR alouattinae OR centrarchiformes OR “common whitethroat” OR “common whitethroats” OR gavialidae OR “grey gurnard” OR “grey gurnards” OR lateolabracidae OR rheiformes OR “tub gurnard” OR “tub gurnards” OR “common chiffchaff” OR “common chiffchaffs” OR garfishes OR “lesser whitethroat” OR “lesser whitethroats” OR myoxidae OR seabasses OR spariformes OR umbridae OR “yellow boxfish” OR anabantiformes OR aotidae OR “common bleak” OR “common bleaks” OR “ common rudd” OR “common rudds” OR “greater pipefish” OR hapale OR nandiniidae OR “stone loaches” OR whinchat OR whinchats OR acanthuriformes OR “brotula barbata” OR “common ling” OR “common lings” OR “common roaches” OR cottonrat OR cottonrats OR douroucoulis OR dromaiidae OR fitches OR fitchew OR galaxiiformes OR laprine OR saimiriinae OR solenette OR tarsii OR “tompot blenny” OR “common dragonet” OR “common dragonets” OR “longspined bullhead” OR “longspined bullheads” OR monotremate OR monotremates OR pempheriformes OR perdicinae OR presbytini OR smegmamorpha OR “bighead gobies” OR “carangaria incertae sedis” OR coiidae OR “fivebeard rockling” OR foulmart OR foumart OR grasskeet OR “greater pipefishes” OR ibices OR millionfish OR muguliformes OR “norwegian topknot” OR peewit OR “red sea sailfin tang” OR rupicapras OR sheatfishes OR “tompot blennies” OR “twait shad” OR “yellow boxfishes”))	24,852,001
#7	(#5) NOT #6	2,863

Abbreviations: SCD, Subjective Cognitive Decline; MCI, Mild Cognitive Impairment; pre-MCI, Pre–Mild Cognitive Impairment; AD, Alzheimer's Disease

**TABLE S3:** Embase Search Strategy Including Full Search Terms (2025 March)

Line	Keywords	Records Retrieved
1	exp middle aged/or exp aged/or exp aged hospital patient/	5496043
2	(old or older adults or senior or elder* or aged).ab,kf,ti.	3469808
3	1 or 2	7662000
4	exp “disorders of higher cerebral function”/	1077705
5	(Cogniti* or dementia or Mild Cognitive Impairment or Neurocognitive Disorder or Subjective Cognitive Decline or Neurocognitive disorders).ab,kf,ti.	946055
6	4 or 5	1581529
7	exp epidemiology/5029303	
8	(incidence or prevalence or occurrence or rate* or epidemiolog* or “general population” or community or population).ab,kf,ti.	9961832
9	7 or 8	1195182
10	Africa.mp.	241438
11	(“subSaharan Africa” or Algeria or Egypt or Libya or Morocco or Tunisia or Cameroon or “Central African Republic” or Chad or Congo or “Democratic Republic of the Congo” or “Equatorial Guinea” or Gabon or Burundi or Djibouti or Eritrea or Ethiopia or Kenya or Rwanda or Somalia or Sudan or South Sudan or Tanzania or Uganda or Angola or Botswana or Lesotho or Mozambique or “Ivory Coast” or Namibia or “South Africa” or Swaziland or Zambia or Zimbabwe or Benin or “Burkina Faso” or “Coôte d'Ivoire” or Gambia or Ghana or Guinea or “Guinea-Bissau” or Liberia or Mali or Malawi or Mauritania or Mauritius or Eswatini or Madagascar or Niger or Nigeria or Senegal or “Sierra Leone” or Togo).ab,kf,ti.	524009
12	10 or 11	643792
13	3 and 6 and 9 and 12	2454

**TABLE S4:** Prevalence of SCD and MCI in Africa: Study Characteristics

Study ID/Reference	Country	Geographical Region	Study Design	Sample Size (n)	Number of Cases	Reported Prevalence	Diagnostic Criteria	Assessment Instruments	Diagnosis	Setting	Population Source	Mean Age (Years)	Female (%)Category	Sampling Technique	Diagnostic Criteria Group
**SCD Studies**															
Ismail et al., 2024	Egypt	North Africa	Cross-sectional	200	107	53.5	Self-experienced decline, intact cognition	ACE-III, RUDAS	SCD	Urban	Community & clinic	65 – 69	<40%	Probability	SCD Criteria
Röhr et al., 2020)	Republic of Congo & CAR	Central Africa	Cross-sectional	1,867	943^*^	50.5	Self-experienced decline, intact cognition	COSMIC Memory questions	SCD	Mixed	Community (≥60)	70 – 74	40 – 60%	Probability	SCD Criteria
Ramlall et al., (2013)	South Africa	Southern Africa	Cross-sectional	251	112^#^	44.6	Self-experienced decline, intact cognition	MMSE, SMRS	SCD	Urban	Community	70 – 74	61 – 80%	Convenience	SCD Criteria
**MCI Studies**															
Abdelaziz et al., 2024	Egypt	Suez Canal Area	Cross-sectional	156	58	37.2	DSM-V	MoCA	MCI	Mixed	Residential Care	65 – 69	40 – 60%	Convenience	Standar dized Diagnostic Frame works
Ismail et al., 2024	Egypt	North Africa	Cross-sectional	200	93	46.5	Cognitive scores (ACE-III: 75 – 81; RUDAS: 19 – 22)	ACE-III, RUDAS	MCI	Urban	Community & clinic	65 – 69	<40%	Probability	Neuropsy chological Test Cutoff Scores
Mukhtar et al., 2024	Nigeria	West Africa	Cross-sectional	143	91	63.6	MoCA score <26	MoCA	MCI	Mixed	Community	65 – 69	40 – 60%	Probability	Neuropsy chological Test CutOff Scores
Tawfik, 2024	Egypt	North Africa	Cross-sectional	470	177	37.7	MoCA cutoff, ASCA	MoCA, ASCA	MCI	Mixed & clinic	Community	65 – 69	<40%	Convenience	Neuropsy chological Test Cutoff Scores
Anieto, 2023	Nigeria	West Africa	Cross-sectional	160	95	59.4	10-WDRT score ≤2	10-WDRT, Katz Index	MCI	Hospital	Clinic	70 – 74	40 – 60%	Probability	Neuropsy chological Test Cutoff Scores
Ucheagwu, 2023	Nigeria	West Africa	Longitudinal cohort	441	57	12.9	Actuarial neuropsycho logical criteria	UDS-3, MoCA	MCI	Mixed	Community	65 – 69	40 – 60%	Convenience	Study-Specific
Fekadu et al., 2022	Ethiopia	East Africa	Cross-sectional	423	178	42.1	MMSE (education-adjusted)	MMSE	MCI	Urban	Community	65 – 69	40 – 60%	Probability	Neuropsy chological Test Cutoff Scores
Gela, 2022	Ethiopia	East Africa	Cross-sectional	393	82	20.9	SMMSE score: 20 – 24	SMMSE	MCI	Urban	Community	60 – 64	40 – 60%	Probability	Neuropsy chological Test Cutoff Scores
Nneamaka et al., 2022	Nigeria	West Africa	Cross-sectional	126	17	13.5	6-CIT score 8 – 14	6-CIT	MCI	Urban	Community	65 – 69	61 – 80%	Probability	Neuropsy chological Test Cutoff Scores
Ofori et al., 2022	Ghana	West Africa	Cross-sectional	5011	2171	43.3	Self-reported memory difficulty	Survey	MCI questions (subjective) (WHO SAGE Wave 1)	Mixed	Community	≥60	40 – 60%	Probability	Self-reported / subjective MCI
Amoo, 2020	Nigeria	West Africa	Cross-sectional	532	11	2.1	Petersen's criteria	MMSE	MCI	Urban	Community	70 – 74	61 – 80%	Probability	MCI / prodromal AD research criteria
Koyanagi et al., 2019a	Ghana	West Africa	Cross-sectional	4,201	311	7.4	NIA-AA	PSS (2-item),	MCI	Mixed	Community	60 – 64	40 – 60%	Probability	MCI / prodromal AD research criteria
Koyanagi et al. (2019)b	South Africa	Sub-Saharan Africa	Cross-sectional	3672	312	8.5	NIA-AA	CERAD word list, WAIS digit span, animal naming	MCI	Mixed	Community	60 – 64	40 – 60%	Probability	MCI / prodromal AD research criteria
Zohoun et al., 2019	CAR	Central Africa	Population-based, two-phase	225	61	27.1	Petersen's criteria	CSI-D, NPI-Q	MCI	Mixed	Community	75 – 79	>80%	Probability	MCI / prodromal AD research criteria
	ROC	Central Africa	Population-based, two-phase	307	52	17.1	Petersen's criteria	CSI-D, NPI-Q	MCI	Mixed	Community	75 – 79	61 – 80%	Probability	MCI / prodromal AD research criteria
Adebiyi et al., 2016	Nigeria	West Africa	Cross-sectional	623	123	19.7	Petersen's criteria	IDEA, DSM-IV,	MCI	Rural	Community	70 – 74	61 – 80%	Convenience	MCI / prodromal AD research criteria
Ogunniyi et al., 2016	Nigeria	West Africa	Cross-sectional	613	111	18.1	Petersen's criteria	IDEA cognitive screen	MCI	Mixed	Community	70 – 74	>80%	Probability	MCI / prodromal AD research criteria
Samba et al., 2016	ROC	Central Africa	Cohort	910	56	5.4	Petersen's criteria	CSI-D, FCSRT	MCI	Mixed	Community	70 – 74	>80%	Probability	MCI / prodromal AD research criteria
Khedr et al., 2015	Egypt	North Africa	Cross-sectional	691	12	1.7	MES score (62 – 75), CDR=0.5	MES, MMSE, WMS-m	MCI	Mixed	Community	65 – 69	40 – 60%	Probability	Neuropsy chological Test Cut-Scores
Paddick et al., 2015	Tanzania	East Africa	Cohort	296	46	7.0	IWG criteria	CSI-D, CERAD 10-word list	MCI	Rural	Community	80 – 84	61 – 80%	Probability	MCI / prodromal AD research criteria
Pilleron et al., 2015	CAR	Central Africa	Cross-sectional	973	72	7.2	Petersen's criteria	CSI-D	MCI	Mixed	Community	70 – 74	61 – 80%	Probability	MCI / prodromal AD research criteria
	ROC	Central Africa	Cross-sectional	1,029	61	6.1	Petersen's criteria	CSI-D	MCI	Mixed	Community	70 – 74	61 – 80%	Probability	MCI / prodromal AD research criteria
Ramlall et al., 2013	South Africa	Southern Africa	Cross-sectional	140	38	27.1	IWG criteria	MMSE	MCI	Urban	Residential Care	75 – 79	61 – 80%	Convenience	MCI / prodromal AD research criteria
Baiyewu et al., 2012	Nigeria	West Africa	Cohort	108	53	49.1	CIND/MCI, MMSE	NPI, BDS, MMSE, DSM m-R	MCI	Urban	Community	80 – 84	>80%	Probability	Study-specific criteria
Khater & Abouelezz, 2011	Egypt	North Africa	Cross-sectional	120	46	38.3	MoCA score <26	MMSE, MoCA	MCI	Urban	Residential Care	70 – 74	61 – 80%	Convenience	Neuropsy chological Test Cut-Off Scores
Guerchet et al., 2010	CAR	Central Africa	Cross-sectional	496	124	25.0	CIND/MCI, CSI-D ≤25.5/30 or FWT ≤10/10	CSI-D, 5-Word Test	MCI	Urban	Community	70 – 74	40 – 60%	Convenience	Study-specific criteria
	ROC	Central Africa	Cross-sectional	520	98	18.8	CIND/MCI, CSI-D ≤25.5/30 or FWT ≤10/10	CSI-D, FWT, DSM-IV, NINCDS-ADRDA	MCI	Urban	Community	70 – 74	61 – 80%	Convenience	Study-specific criteria
Guerchet et al., 2009	Benin	West Africa	Cross-sectional	502	52	10.4	CIND/MCI, CSI-D ≤25.5/30 or FWT ≤10/10	CSI-D, FWT, DSM-IV, NINCDS-ADRDA	MCI	Rural	Community	80 – 84	61 – 80%	Convenience	Study-specific criteria
Rahman & El Gaafary, 200$	>Egypt	North Africa	Cross-sectional	268	104	38.8	MoCA score <26	MoCA, CAMCOG	MCI	Urban	Community	65 – 69	40 – 60%	Probability	Neuropsy chological Test Cut-Off Scores

Notes

* Number of Cases: 943 (based on Criterion 1: endorsement of self-experienced decline without objective impairment),

# Derived SCD cases = 112 (37% of total sample; 44.6% of cognitively intact subgroup).

Abbreviations SCD: Subjective Cognitive Decline; MCI: Mild Cognitive Impairment; MoCA: Montreal Cognitive Assessment; ASCA: Arabic Scale for Cognitive Assessment; 10-WDRT: 10-Word Delayed Recall Test; SMMSE: Standardized Mini-Mental State Examination; NIA-AA: National Institute on Aging–Alzheimer's Association; CERAD: Consortium to Establish a Registry for Alzheimer's Disease; WAIS: Wechsler Adult Intelligence Scale; PSS: Perceived Stress Scale; CSI-D: Community Screening Instrument for Dementia; FCSRT: Free and Cued Selective Reminding Test; MES: Memory and Executive Screening; MMSE: Mini-Mental State Examination; WMS-III: Wechsler Memory Scale-III; IWG: International Working Group; NPI: Neuropsychiatric Inventory; NPI-Q: Neuropsychiatric Inventory Questionnaire; BDS: Blessed Dementia Scale; CIND: Cognitive Impairment No Dementia; FWT: Five-Word Test; CAMCOG: Cambridge Cognitive Examination; ADL: Activities of Daily Living; IADL: Instrumental Activities of Daily Living; DSM-IV: Diagnostic and Statistical Manual of Mental Disorders, 4th Edition; DSM-5: Diagnostic and Statistical Manual of Mental Disorders, 5th Edition; NINCDS-ADRDA: National Institute of Neurological and Communicative Disorders and Stroke–Alzheimer's Disease and Related Disorders Association; NINDS-AIREN: National Institute of Neurological Disorders and Stroke–Association Internationale pour la Recherche et l'Enseignement en Neurosciences; UDS-3: Uniform Data Set-Version 3; 6-CIT: 6-Item Cognitive Impairment Test; GDS: Geriatric Depression Scale; WHOQOL-BREF: World Health Organization Quality of Life-BREF; FAST: Functional Assessment Staging Tool; ACE-III: Addenbrooke's Cognitive Examination-III; RUDAS: Rowland Universal Dementia Assessment Scale; ADLQ: Activities of Daily Living Questionnaire; CAR: Central African Republic; ROC: Republic of Congo; COSMIC: Cohort Studies of Memory in an International Consortium

**TABLE S5:** Sociodemographic and Clinical Features of Participants with Mild Cognitive Impairment Across Africa

Author (Year)	Married %	Education Low %	Education Med %	Education High %	Smoking Ever %	DM Yes %	HTN Yes %	Family History Yes %
Abdelaziz et al. (2024)	31.0	10.3	65.5	24.1	74.1	34.5	58.6	20.7
Ismail et al. (2024)	84.5	17.5	NR	82.5	NR	NR	NR	11.5
Mukhtar et al. (2024)	58.7	70.6	NR	29.4	NR	NR	NR	NR
Tawfik (2024)	67.9	58.5	NR	41.5	47.2	64.2	56.1	45.8
Anieto (2023)	54.4	13.7	44.4	41.9	20.7	NR	62.5	NR
Ucheagwu (2023)	NR	62.1	23.6	25.4	NR	NR	NR	NR
Fekadu et al. (2022)	52.2	61.5	23.4	15.1	2.4	18.0	30.7	8.0
Gela et al. (2022)	94.0	21.6	59.1	19.3	0.5	NR	NR	NR
Nneamaka et al. (2022)	67.5	4.0	61.1	34.9	31.0	0.0	52.4	22.2
Ofori et al. (2022)	NR	50.3	34.7	27.2	NR	NR	NR	NR
Amoo (2020)	43.6	61.7	33.4	4.9	NR	10.5	37.8	17.5
Koyanagi et al. (2019a)	NR	NR	NR	NR	NR	NR	NR	NR
Koyanagi et al. (2019b)	NR	NR	NR	NR	33.4	9.1	78.6	NR
Zohoun et al. (2019)	19.9	89.0	NR	11.0	37.8	4.4	53.8	NR
Zohoun et al. (2019)	25.3	87.5	NR	12.5	12.6	10.7	67.8	NR
Adebiyi et al. (2016)	46.1	69.3	25.4	5.2	12.4	2.7	NR	NR
Ogunniyi et al. (2016)	NR	86.5	NR	13.5	NR	NR	NR	NR
Samba et al. (2016)	17.9	87.5	NR	1.8	NR	8.9	60.7	NR
Khedr et al. (2015)	NR	66.7	NR	NR	NR	16.7	33.3	NR
Paddick et al. (2015)	NR	55.5	44.5	NR	NR	4.3	69.6	NR
Pilleron et al. (2015)	34.2	69.2	NR	NR	31.8	NR	NR	NR
Pilleron et al. (2015)	38.9	68.4	NR	NR	12.9	NR	NR	NR
Ramlall et al. (2013)	NR	75.7	NR	7.9	63.2	21.1	57.9	10.5
Baiyewu et al. (2012)	NR	92.5	NR	7.5	NR	NR	NR	NR
Khater & Abouelezz (2011)	NR	28.3	56.6	15.2	30.4	30.4	45.7	NR
Guerchet et al. (2010)	41.1	56.7	NR	43.3	36.6	6.3	56.3	NR
Guerchet et al. (2010)	38.1	48.8	NR	51.2	32.5	21.9	72.3	NR
Guerchet et al. (2009)	34.6	98.1	1.9	0	57.7	23.1	28.8	NR
Rahman & El Gaafary (2009)	42.0	52.0	44.6	17.1	64.2	NR	NR	NR

Abbreviations: DM, diabetes mellitus; HTN, hypertension; NR, not reported; % = percent.

**TABLE S6:** Sociodemographic and Clinical Features of Participants with Subjective Cognitive Decline Across Africa

Author (Year)	Married %	Years of Education mean (± SD)	Smoking Ever %	DM Yes %	HTN Yes %	Family History Yes %
Ismail et al. (2024)	84.5	11.7 (± 5.9)	NR	NR	NR	11.5
Röhr et al., 2020)	NR	2.06 (3.86)	NR	NR	NR	NR
Ramlall et al., (2013)	NR	9.4 ± 1.7	NR	NR	NR	NR

Abbreviations: SD, standard deviation; DM, diabetes mellitus; HTN, hypertension; NR, not reported.

**TABLE S7:** Publication Bias and Heterogeneity Assessment for SCD Prevalence

Metric	Value
I^2^ (%)	27.0
τ^2^	0.0018
H^2^	NA
Egger's Test *p-*value	NA

Abbreviations: I^2^, inconsistency index; τ^2^, between-study variance; H^2^, heterogeneity ratio; NA, not applicable.

**TABLE S8:** Comprehensive Sensitivity Analysis Summary for SCD studies

Analysis	N_Studies	Pooled_Prevalence	CI_Lower	CI_Upper	I2_Percent
Main Analysis	3.0000	0.4968	0.4014	0.5924	50.5985
Smallest Study Removed	2.0000	0.4829	0.1792	0.7999	67.2963
Largest Study Removed	2.0000	0.4891	0.0907	0.9019	71.4527
Alternative Tau (REML)	3.0000	0.4968	0.4019	0.5920	50.5985

Abbreviations: CI, confidence interval; I^2^, inconsistency index; REML, restricted maximum likelihood.

**TABLE S9:** Publication Bias and Heterogeneity Assessment for MCI Prevalence

Metric	Value	Interpretation
I^2^ (%)	99.1	% of total variation due to heterogeneity
τ^2^	1.3984	Between-study variance
Egger's Test *P* value	.2949	Test for funnel plot asymmetry

Abbreviations: I^2^, inconsistency index; τ^2^, between-study variance; *P* value, probability value (Egger's test).

**TABLE S10:** Comprehensive Sensitivity Analysis Summary for MCI

Analysis	N_Studies	Pooled_Prevalence	CL_Lower	CL_Upper	I2_Percent
Main Analysis	29.0000	0.2003	0.1376	0.2823	99.1311
Smallest Study Removed	28.0000	0.1928	0.1313	0.2739	99.1529
Largest Study Removed	28.0000	0.2071	0.1415	0.2927	98.9725
Alternative Tau (REML)	29.0000	0.2003	0.1376	0.2823	99.1311

Key to abbreviation: CL, confidence limit.

**TABLE S11:** Detailed Country-Level Subgroup Analyses and Full Multivariable Meta-Regression

Subgroup Analyses	No. of Studies	Prevalence, 95% CI (%)	I2 (%)		*P* Values Within Subgroups	*P* Values Across Subgroups
Country-Level Subgroup Results						
Country						.8450
Central African Rep.	3	16.92 (6.61–36.94)	98.1		.0032	
Congo	4	10.37 (5.26–19.45)	96.8		<.001	
Egypt	6	27.33 (16.34–42.01	96.7		.0035	
Ethiopia	2	30.50 (13.98–54.24	97.6		.1042	
Ghana	2	23.94 (2.10–82.19)	99.9		.3988	
Nigeria	8	23.38 (12.50–39.47	98.0		.0022	
South Africa	2	15.53 (4.50–41.76)	97.9		.0147	
Meta-regression analyses						
**Predictor**	**Coefficient**	**Standard Error**	**95% Lower CL**	**95% Upper CL**	**z_value**	***P* Value**
Intercept	-4.3267	2.9032	-10.0169	1.3635	-1.49	.1361
Married (%)	0.0463	0.0187	0.0097	0.0829	2.48	.0131
Low Education (%)	-0.0138	0.0133	-0.0399	0.0123	-1.04	.2990
Medium Education (%)	-0.0214	0.0229	-0.0662	0.0235	-0.93	.3510
High Education (%)	-0.0041	0.0207	-0.0447	0.0365	-0.20	.8433
Ever Smoked (%)	-0.0016	0.0125	-0.0262	0.0230	-0.13	.8987
Diabetes Mellitus (%)	0.0530	0.0235	0.0069	0.0991	2.25	.0243
Hypertension (%)	0.0517	0.0217	0.0091	0.0942	2.38	.0173
Family History (%)	-0.0348	0.0276	-0.0888	0.0193	-1.26	.2074
Age: 60–64	-3.2169	1.0719	-5.3178	-1.1159	-3.00	.0027
Age: 65–69	-1.1906	0.9085	-2.9711	0.5900	-1.31	.1900
Age: 70–74	-0.9093	0.9725	-2.8154	0.9968	-0.93	.3498
Age: 75–79	0.3325	1.1663	-1.9534	2.6183	0.29	.7756
Age: 80–84	-0.1252	1.1282	-2.3365	2.0861	-0.11	.9116
Female (%)>80	1.4811	1.8447	-2.1344	5.0965	0.80	.4220
Female (%)40–60	1.3275	1.3464	-1.3113	3.9664	0.99	.3241
Female (%)61–80	-0.0300	1.5887	-3.1438	3.0838	-0.02	.9849

Key to abbreviations: CL, confidence limit

**TABLE S12:** Variance Inflation Factors (VIF) for Predictors Included in Multivariable Meta-Regression of MCI Prevalence

Predictor_Display	VIF
Married (%)	2.34
Low Education (%)	2.85
Medium Education (%)	1.95
High Education (%)	2.11
Ever Smoked (%)	1.44
Diabetes Mellitus (%)	2.87
Hypertension (%)	1.70
Family History (%)	2.65

**TABLE S13:** JBI Critical Appraisal Checklist for Studies Reporting Prevalence Data using the Joanna Briggs Institute's Checklist for Prevalence Studies.

Study ID	Author	Year	Record Number	Q1	Q2	Q3	Q4	Q5	Q6	Q7	Q8	Q9	Overall Appraisal	Comments
**SCD Studies**														
1	Ismail et al.	2024	1	Yes	Yes	Yes	Yes	Yes	Yes	Yes	Yes	Yes	Include	High quality (9/9 = 100% “Yes”).
2	Röhr et al.	2020	2	Yes	Yes	Yes	Yes	Yes	Yes	Yes	Yes	Unclear	Include	High quality (8/9 = 88.89% “Yes”).
3	Ramlall et al.	2013	3	Yes	Yes	Unclear	Yes	Yes	Yes	Yes	Yes	Unclear	Include	High quality (7/9 = 77.78% “Yes”).
**MCI Studies**														
4	Abdelaziz et al.	2024	4	Yes	No	Yes	Yes	Yes	Yes	Yes	Yes	Unclear	Include	High quality (7/9 = 77.78% “Yes”).
5	Ismail et al.	2024	5	Yes	Yes	Yes	Yes	Yes	Yes	Yes	Yes	Yes	Include	High quality (9/9 = 100% “Yes”).
6	Mukhtar et al.	2024	6	Yes	Yes	Yes	Yes	Yes	Yes	Yes	Yes	Partial	Include	High quality (8/9 = 88.89% “Yes”).
7	Tawfik et al.	2024	7	Yes	No	Yes	Yes	Yes	Yes	Yes	Yes	No	Include	High quality (7/9 = 77.78% “Yes”).
8	Anieto et al.	2023	8	Yes	No	Yes	Yes	Yes	Yes	Yes	Yes	No	Include	High quality (7/9 = 77.78% “Yes”).
9	Ucheagwu et al.	2023	9	Yes	Probably yes	Yes	Yes	Yes	Yes	Yes	Yes	Yes	Include	High quality (8/9 = 88.89% “Yes”).
10	Fekadu et al.	2022	10	Yes	Yes	Partial	Yes	Yes	Yes	Partial	Yes	Yes	Include	Moderate-High (7/9 = 77.78% “Yes”).
11	Gela et al.	2022	11	Yes	Yes	Yes	Yes	Yes	Yes	Yes	Yes	No	Include	High quality (8/9 = 88.89% “Yes”).
12	Nneamaka et al.	2022	12	Yes	Yes	Yes	Yes	Yes	Yes	Yes	Yes	Yes	Include	High quality (9/9= 100% “Yes”).
13	Ofori et al.	2022	32	Yes	Yes	Yes	Partial	Yes	No	Partial	Yes	N/A	Include	Moderate quality (5/9 = 55.56% “Yes”).
14	Amoo et al.	2020	14	Yes	Yes	Yes	Yes	Yes	Yes	Yes	Yes	No	Include	High quality (8/9 = 88.89% “Yes”).
15	Koyanagi et al.a	2019	15	Yes	Yes	Yes	Yes	Yes	Yes	Yes	Yes	Yes	Include	High quality (9/9 = 100% “Yes”).
16	Koyanagi et al.b	2019	16	Yes	Yes	Yes	Yes	Yes	Yes	Yes	Yes	Yes	Include	High quality (9/9 = 100% “Yes”).
17	Zohoun et al.	2019	17	Yes	Yes	Yes	Yes	Yes	Yes	Yes	Yes	Yes	Include	High quality (9/9 = 100% “Yes”).
18	Adebiyi et al.	2016	18	Yes	Unclear	Yes	Yes	Yes	Yes	Yes	Yes	Unclear	Include	High quality (7/9 = 77.78% “Yes”).
19	Ogunniyi et al.	2016	19	Yes	Yes	Yes	Yes	Yes	Yes	Yes	Yes	Yes	Include	High quality (9/9= 100% “Yes”).
20	Samba et al.	2016	20	Yes	Yes	Yes	Yes	Yes	Yes	Yes	Yes	Yes	Include	High quality (9/9= 100% “Yes”).
21	Khedr et al.	2015	21	Yes	Yes	Yes	Yes	Yes	Yes	Yes	Yes	Unclear	Include	High quality (8/9 = 88.89% “Yes”).
22	Paddick et al.	2015	22	Yes	Yes	Yes	Yes	Yes	Yes	Yes	Yes	Yes	Include	High quality (9/9 = 100% “Yes”).
23	Pilleron et al.	2015	23	Yes	Yes	Yes	Yes	Yes	Yes	Yes	Yes	Yes	Include	High quality (9/9 = 100% “Yes”).
24	Ramlall et al.	2013	24	Yes	Yes	Unclear	Yes	Yes	Yes	Yes	Yes	Unclear	Include	High quality (7/9 = 77.78% “Yes”).
25	Baiyewu et al.	2025	25	Yes	Yes	No	Yes	Yes	Yes	Yes	Yes	Unclear	Include	High quality (7/9 = 77.78% “Yes”).
26	Khater & Abouelezz.	2011	26	Yes	Unclear	Yes	Yes	Yes	Yes	Yes	Yes	Unclear	Include	High quality (7/9 = 77.78% “Yes”).
27	Guerchet et al.	2010	27	Yes	Yes	Yes	Yes	Yes	Yes	Yes	Yes	Yes	Include	High quality (9/9 = 100% “Yes”).
28	Guerchet et al.	2009	28	Yes	Yes	Yes	Yes	Yes	Yes	Yes	Yes	Yes	Include	High quality (9/9 = 100% “Yes”).
29	Rahman & El Gaafary.	2009	29	Yes	Yes	Yes	Yes	Yes	Yes	Yes	Yes	Yes	Include	High quality (9/9 = 100% “Yes”).

Notes: High quality = ≥70% of items rated “Yes”, Moderate quality = often taken as 50 to 69% “Yes”, Low quality = <50% “Yes”

Q1: Was the sample frame appropriate?

Q2: Were participants sampled appropriately?

Q3: Was the sample size adequate?

Q4: Were subjects & setting described in detail?

Q5: Was data analysis conducted with sufficient coverage?

Q6: Were valid methods used for condition identification?

Q7: Was the condition measured reliably for all participants?

Q8: Was there appropriate statistical analysis?

Q9: Was the response rate adequate / managed appropriately?
